# Exposure patterns among Coast Guard responders to the Deepwater Horizon Oil Spill: A latent class analysis

**DOI:** 10.1097/EE9.0000000000000211

**Published:** 2022-05-02

**Authors:** Matthew O. Gribble, Taj Keshav, Hristina Denic-Roberts, Lawrence S. Engel, Jennifer A. Rusiecki

**Affiliations:** a Department of Epidemiology, University of Alabama at Birmingham, Birmingham, Alabama; b Department of Preventive Medicine and Biostatistics, Uniformed Services University, Bethesda, Maryland; c Oak Ridge Institute for Science and Education, Bethesda, Maryland; d Department of Epidemiology, Gillings School of Public Health, University of North Carolina, Chapel Hill, North Carolina

**Keywords:** Exposure science, Structural equation modeling, Occupational health, Latent variables, Disaster response

## Abstract

**Background:**

The Deepwater Horizon Oil Spill was an environmental crisis for which multiple groups, including the United States Coast Guard (USCG), provided emergency response services. A cohort of 5,665 USCG oil spill responders completed postdeployment surveys eliciting information on a variety of topics, including oil spill–related exposures and experiences. Our objective was to determine the most common exposure patterns among USCG responders.

**Methods:**

We used latent class analysis based on six indicator variables reflecting different aspects of the responders’ experiences: exposure to oil, exposure to engine exhaust fumes or carbon monoxide, hand sanitizer use, sunblock use, mosquito bites, and level of anxiety. We validated our interpretation of these latent classes using ancillary variables.

**Results:**

The model distinguished four distinct exposure profiles, which we interpreted as “low overall exposure” (prevalence estimate = 0.18), “low crude oil/exhaust and moderate time outdoors/anxiety (prevalence estimate = 0.18), “high crude oil/exhaust and moderate time outdoors/anxiety” (prevalence estimate = 0.25), and “high overall exposure” (prevalence estimate = 0.38). The validation analysis was consistent with our interpretation of the latent classes.

**Conclusions:**

The exposure patterns identified in this analysis can help inform future studies of the health impacts of exposure mixtures among USCG oil spill responders.

**What this study adds**

This study used a latent class analysis modeling approach to characterize the overall patterns of exposure, considering multiple exposure domains, that were commonly reported on surveys by Coast Guard responders to the Deepwater Horizon Oil Spill. The latent class model suggested four prevailing exposure patterns in this responder population.

## Introduction

The United States Coast Guard (USCG) deployed personnel in response to the Deepwater Horizon oil spill (DWH), a disaster in 2010 wherein the explosion of the Deepwater Horizon offshore drilling rig in the Gulf of Mexico caused one of the largest oil spills in US history.^1^ There were 87 days of continuous oil discharge before effective well-capping, resulting in an unprecedented amount of oil spilling into the Gulf. There were over 47,000 response personnel at the peak of response operations during the summer of 2010, with nearly 9,000 from the USCG.^1^

The Deepwater Horizon Oil Spill Coast Guard Cohort (DWH-CG) is a prospective cohort study designed to assess the potential acute and long-term health effects of exposures encountered during USCG response efforts.^2^ Previous research conducted with this cohort identified positive associations of crude oil and oil dispersant exposure with acute respiratory,^2,3^ neurological,^2–4^ and dermal^2^ symptom onset, as well as with longer term respiratory and dermal health outcomes.^2^ These findings are consistent with other literature on the health effects of oil spill exposure,^5,6^ and with findings of impaired lung function and respiratory outcomes investigated in another prospective cohort study of DWH response workers, the Gulf Long-Term Follow Up (GuLF) Study.^7–9^ In addition, previous studies reported novel associations of crude oil exposure with acute gastrointestinal and genitourinary symptoms in the DWH-CG study,^2^ as well as associations between heat exposure and heat-related symptoms during the DWH response.^10^

While associations between specific exposures (e.g., exposure to oil) have been considered individually in relation to health outcomes, this approach is limited. There are myriad environmental exposures encountered during an oil spill response, and although several of the exposures have been assessed for epidemiological relevance, other less well-studied exposures could also be contributing to health outcomes. Identifying the most problematic under-studied exposures is an important challenge for environmental epidemiology and one which if addressed as an agnostic search can be like finding the proverbial “needle in the haystack”: inefficient and intractable without enormous resources. The search might be more efficient by identifying informative subsets of the population who are discordant both on health outcomes and on a close surrogate of the unknown exposure.^11^ The main goal of this study was to provide measurement of an exposure surrogate for a suite of unmeasured exposures potentially important for health outcomes following an oil spill disaster response.

The set of environmental exposures encountered during a disaster response is structured both by the actions of the responders and by the environments in which the responders act. We therefore expected that exposure patterns in the DWH Oil Spill Coast Guard Cohort population would be clustered, to the extent that persons had similar experiences during the disaster response efforts. These clusters could be useful for informing more efficient epidemiological studies evaluating novel risk factors, because the clusters are reasonable surrogates for multiple unmeasured exposures.

In this study, we describe the development of a latent class model to characterize common patterns of lived experience during the spill response and concomitant environmental exposures in the DWH-CG Study.

## Methods

### Study population

The DWH-CG study population has been described previously.^2^ Briefly, the full cohort includes 8,696 oil spill responders and 44,823 other Coast Guard personnel who did not respond to the DWH oil spill (nonresponders), and categorizes personnel as active duty or in the Selected Reserve between 20 April and 17 December 2010. Of the oil spill responders included in the cohort, 65% (N = 5,665) completed at least one of two surveys administered at the end of their DWH deployment (hereafter referred to as “exit surveys”), which gathered data on exposures and symptoms experienced during their oil spill deployment. We included all 5,665 responders who completed a survey in the current latent class analysis. The responder exposure survey participants had a similar sex distribution and race distribution to the nonparticipants.^2^

This study was approved by the Institutional Review Boards (IRB) of the Uniformed Services University (USU), the USCG, and the University of North Carolina, Chapel Hill. A waiver for informed consent was approved by the USU IRB.

### Exposure data

Two exit surveys were conducted: survey 1, launched on 25 June 2010, and survey 2, launched on 1 November 2010. Both surveys asked responders about deployment-related factors such as duration and timing, location, mission category, exposures to crude oil/oily water, burning crude oil, oil dispersants, combustion engine exhaust, animal/insect bites, personal protective equipment (PPE) use, acute symptoms and injuries, and lifestyle factors. In this study, “mission category” denotes various duties carried out by USCG personnel during deployment. In general, many of the factors assessed were similar between the two surveys. However, survey 1 assessed exposures on an ever/never scale, and survey 2 assessed frequency of exposures using a 5-point scale (1 = never, 2 = rarely, 3 = sometimes, 4 = most of the time, 5 = all of the time). To maximize sample size for statistical analysis, we harmonized survey 1 and 2 responses by limiting the scope of this study to common variables between the two surveys, and dichotomizing survey 2 responses on an ever/never scale. All survey 2 responses graded above a 1 on the 5-point scale were coded as “ever,” and those graded as a 1 were coded as “never.”

### Statistical analysis

#### Selection of candidate indicators from exposure domains

We considered 54 candidate exposure variables, or “candidate indicators” across an initial grouping of seven broad categories of exposure, or “exposure domains,” for inclusion as indicator variables in the latent class analysis (LCA) based on what we deemed most relevant to the exposure scenarios of the USCG responders. The exposure domains were (1) sleep, (2) insect and/or animal bites, (3) smoking, (4) exposure to crude oil, exhaust fumes or carbon monoxide exposure through any exposure route, (5) injury and subsequent care-seeking experiences, (6) anxiety and other psychosocial stressors, and (7) use of PPE (Table S1; http://links.lww.com/EE/A188).

**Figure 1.**
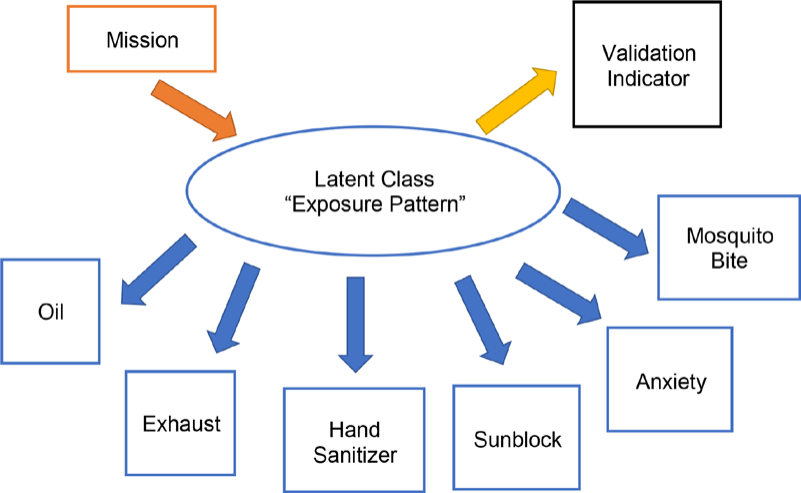
Relationships between variables.

To select the set of indicators used for latent class estimation from across the exposure domains, the following criteria were used: (1) each indicator had to be of a high enough prevalence within the DWH Oil Spill Coast Guard Cohort, to help ensure that the final joint contingency table of indicator combinations would have low data sparsity; (2) the indicators were sampled so to include multiple exposure domains in our latent class definition; and (3) the indicators were selected such that we would not expect strong residual relationships between indicators to exist within each of the finally estimated latent classes (Figure 1). This approach increased the content validity for our latent class enumeration, as the different survey response patterns across the final set of indicators would more broadly capture the range of exposure experiences of DWH responders while minimizing correlated residual errors among the indicators.^12^ These residual errors, if present, could bias model output during latent class analysis.

To confirm there would not be too much extrapolation over sparse data in the latent class model estimation, we looked at the full joint contingency table of the response patterns of the candidate indicator set (Figure S1; http://links.lww.com/EE/A188). Our final model included six highly prevalent indicators with at least one observation for each possible exposure combination (e.g., 2^6^= 64 strata with n > 0 per stratum): (1) ever exposure to crude oil, (2) ever exposure to exhaust fumes or carbon monoxide, (3) hand sanitizer use, (4) sunscreen use, (5) experience of mosquito bites, and (6) experience of anxiety during deployment. No indicators from the domains of sleep, smoking, or injury and subsequent care-seeking experiences were selected for the latent class enumeration. It is possible that residual association between crude oil and exhaust fumes or carbon monoxide conditional on latent class may or may not influence latent class estimation in this application.^12^

#### Enumeration of latent classes for the latent class measurement model

LCA methods classify people into different subgroups based on shared sets of indicator response patterns.^13^ We leverage LCA in our study to classify participants into different subgroups of exposure experiences based on how their self-reported responses to the six candidate survey indicators cluster together. The latent class measurement model we applied used a series of jointly estimated logistic regression models to predict each of the six selected binary indicators as a function of latent class membership. We used the six selected indicators to fit 2-class, 3-class, 4-class, and 5-class measurement models. Goodness-of-fit for each latent class measurement model was assessed via entropy metrics,^14^ Akaike Information Criterion (AIC),^15^ and Bayesian Information Criterion (BIC).^16^ Estimation of the number of latent classes was done before inclusion of any covariates into the model.^17^ Although we assumed independence among the DWH study participants for the purpose of latent class estimation, it is possible that due to the spatiotemporal clustering of participants (e.g., shared service locations, timing of deployment to each location), which were inadequately measured, some residual clustering remained. This could have led to an unmodeled multilevel data structure, and therefore an over-estimation of classes by fit statistics.^18^ Therefore, in addition to quantitative fit metrics, we also used our own judgment to evaluate model reasonableness and validated our interpretation of the preferred model (favored by BIC and judgment of the investigative team) using tests for convergent construct validity,^19^ which led us to prioritize a 4-class model.

#### Construct validity: validation of latent class measurement model by other indicators using constrained models

As a validation of our interpretation of the four-class latent class measurement model, we examined the relationship of the latent classes to other measured variables whose relationships to the latent classes were predictable (i.e., expected a priori) using logistic regression with the latent class predicting the new validation indicator variable. The validation indicators were selected on the subjective basis of our ability to understand them well enough to make a priori predictions of how those should be related to our latent classes if we are right about how we are interpreting the latent classes. Our test for convergent construct validity was performed in two stages. First, we constrained the four-class measurement model by fixing the class probabilities between the six latent class measurement model indicators and each of the four latent classes. In the next step, we added one additional measured “validation variable” as another latent class indicator which was allowed to be freely estimated while the six “model-defining” indicators were constrained. The validation indicators we considered were spending at least some time outdoors, use of personal floatation equipment, Camelbak use, use of bug spray, use of nitrile gloves, and use of Tyvek suit. A separate model was fitted for each of these validation indicators.

#### Regression of latent classes on deployment mission categories

We expected that the clustering of reported exposures among responders to the Deepwater Horizon Oil Spill would be a function of the kind of work done by the responders to the oil spill. Data on the deployment missions performed by the USCG responders were available for all study participants. These missions reflect the general tasks responders were involved in and have been described previously.^2^ Responders reported being involved in up to 27 different missions. To capture the overall oil spill response clean-up experience of each responder, individual missions and mission combinations were evaluated by a former Coast Guard industrial hygienist and two of the authors (J.A.R. and H.D.-R.) with regard to crude oil exposure opportunity. The unique mission combinations of each responder were categorized as missions with lower crude oil exposure opportunity (administrative-like missions), medium crude oil exposure opportunity (low oil/mixed missions), and higher crude oil exposure opportunity (oil-related missions). To test the relationships between mission and latent classes, we initially added mission category as a three-level covariate predicting the latent classes of our four-class, six-indicator measurement model in a jointly estimated unconstrained model (Table S2; http://links.lww.com/EE/A188). However, that model which jointly estimated the relationship of mission to latent class, and of latent class to the model defining indicators, had several parameters (i.e., indicator probabilities given class membership) at the boundary values (e.g., probability equals zero or probability equals one), suggesting unstable estimation. To stabilize model estimation, we constrained the relationships between the four latent classes and six indicators (at the point estimates from the latent class model without a covariate) and only allowed the relationship of mission to the latent class to be flexibly estimated. We report the constrained model output in our main results, but also provide the flexibly estimated model output in our Supplementary Materials; http://links.lww.com/EE/A188. We calculated odds ratios (OR) and 95% confidence intervals (CI) using the administrative-like mission category as the reference group.

#### Software

Descriptive data analysis (e.g., prevalence of exposures) was conducted using Stata 14.2 S/E (StataCorp, College Station, TX). Latent class models were estimated using MPlus version 7.4 (Múthen & Múthen, Los Angeles, CA).

## Results

### Selected candidate indicators: response patterns and assessment of data sparsity

Individual response frequencies for candidate indicators considered for the latent class measurement model are reported in Table S1 and Figure S1; http://links.lww.com/EE/A188. The 5,665 survey respondents varied in their exposure to the six indicator variables used in latent class estimation: 49% reported ever having crude oil exposure, 34% reported ever having exhaust fume or carbon monoxide exposure, 37% reported ever being bitten by a mosquito, 14% reported ever experiencing anxiety, 34% reported ever using sunscreen, and 37% reported ever using hand sanitizer. Across these six indicators, there was at least one observation for all 64 possible item response patterns for any given DWH survey respondent (Figure S1; http://links.lww.com/EE/A188). Twelve of the 64 response patterns had fewer than 10 observations, with the sparsest response pattern reported by two survey respondents whose response pattern was being exposed to crude oil exposure, never exposed to exhaust fumes or carbon monoxide, ever bitten by a mosquito, ever experienced anxiety, never used sunscreen, and never used hand sanitizer.

### Latent class measurement model

We considered several six-indicator latent class models with the number of latent classes ranging from 2 to 5. The four-class model had the lowest BIC of the models considered (Table 1). The entropy was equivalent (0.63) for the three-, four-, and five-class models, but higher for the two-class model (0.78), indicating that the two-class model was most distinctly able to sort participants into “high” and “low” exposure categories, and more nuanced groupings (i.e., a higher number of latent classes) made it more challenging to sort participants as distinctly. Despite this, we prioritized the four-class model since it would potentially distinguish a greater number of exposure pattern subtypes than the two- or three-class models, while being more parsimonious (with a lower BIC) than the five-class model that had the same entropy. We therefore used the four-class model in subsequent validation analyses and in relation to the mission covariate.

**Table 1.** Model fitting diagnostics.

	2 Class	3 Class	4 Class	5 Class
Entropy	0.78	0.63	0.63	0.63
AIC	36,974	36,656	36,369	36,353
BIC	37,060	36,788	36,548	36,578

AIC indicates Akaike Information Criterion; BIC, Bayesian Information Criterion.

The probabilities for each of the six indicators within each class of the four-class model are reported in Table 2. Estimated class probabilities of crude oil exposure, exhaust fume or carbon monoxide exposure, mosquito bite, and experience of anxiety had generally monotonic increases across these four latent classes. We labeled the four latent classes as reflecting four possible exposure experiences of the DWH responders: “low overall exposure,” “low crude oil/exhaust exposure with moderate outdoor time/anxiety,” “high crude oil/exhaust exposure with moderate outdoor time/anxiety,” and “high overall exposure.” For the purposes of our labeling of the classes, the “outdoor time” label was based on how strongly hand sanitizer use, sunblock use, and mosquito bites loaded onto the classes, as these indicators are reflective of either PPE (hand sanitizer, sunblock) or other exposures (mosquito bites) that DWH responders experienced when they worked outdoors. The middle two classes in the four-class model were also distinguished by their probabilities of hand sanitizer use, sunblock, and mosquito bite: persons in the second class had higher hand sanitizer and sunblock use and lower probability of mosquito bite, than persons in the third latent class, but probability of sunblock use and mosquito bites in both suggested moderate time outdoors relative to the other classes.

**Table 2.** Exposure profiles in the Deepwater Horizon Oil Spill Coast Guard Cohort Study (n = 5,665).

Binary exposure (yes/no)	Class 1 (~18%)“low overall exposure”	Class 2 (~18%)“low crude oil/exhaust exposure with moderate outdoor time/anxiety”	Class 3 (~25%)“high crude oil/exhaust exposure with moderate outdoor time/anxiety”	Class 4 (~38%)“high overall exposure”
Any crude oil exposure	0.05	0.20	0.61	0.83
Exhaust fumes or carbon monoxide	0.06	0.20	0.95	0.97
Hand sanitizer	0.12	0.69	0.47	0.94
Sunblock	0.11	0.78	0.51	0.96
Mosquito bite	0.10	0.47	0.69	0.92
Anxiety	0.05	0.11	0.15	0.20

Probability of self-reporting “yes” to each exposure, occurring at any time, among members of each latent class, is shown in cells. Estimated prevalence of each latent class across the population is shown in parentheses.

### Construct validity of the 4-class measurement model

Class probabilities for each of the six indicators within the four-class measurement model were constrained to the probabilities reported in Table 2, and a seventh validation indicator was added to assess construct validity. The resulting estimates of class probabilities for different validation indicators are reported in Table 3. For the “low overall exposure” experience latent class, low class probabilities were reported across each of the validation variables: personal flotation equipment use, Camelbak use, bug spray use, nitrile glove use, and Tyvek suit use. Similarly, the “high overall exposure” latent class had the highest class probabilities for each of these validation variables. For the intermediate latent classes, Camelbak use and bug spray use distinguished these two classes. Persons with latent class membership to class 2 “low crude oil/exhaust exposure with moderate outdoor time/anxiety” had a higher class probability of bug spray (53%) than those with latent class membership to class 3 “high crude oil/exhaust exposure with moderate outdoor time/anxiety” (10%). The difference in class probability for CamelBak use was not as strong between those in class 2 (18%) and class 3 (5%).

**Table 3.** Checks of construct validity (n = 5,665).

Validation variable	Class 1 (~18%)“low overall exposure”	Class 2 (~18%)“low crude oil/exhaust exposure with moderate outdoor time/anxiety”	Class 3 (~25%)“high crude oil/exhaust exposure with moderate outdoor time/anxiety”	Class 4 (~38%)“high overall exposure”
Outdoors at least sometimes	0.34	0.63	0.67	0.97
Personal floatation equipment use	0.03	0.54	0.41	0.91
CamelBak use	<0.01	0.18	0.05	0.39
Bug spray use	<0.01	0.53	0.10	0.80
Nitrile gloves use	<0.01	0.26	0.21	0.83
Tyvek suit use	<0.01	0.05	0.04	0.25

Probability of self-reporting “yes” to exposure to each of the validation variables, among members of each latent class. Models were fit separately to allow a new “validation indicator” each, augmenting the same underlying (constrained) latent classes defined per Table 2.

### Regression of latent class on deployment mission categories

Associations between mission categories and latent classes, from the model where latent classes were predefined (i.e., relationships of latent class indicators to the latent classes are fixed while the association of the mission covariate with latent class was freely estimated) are reported in Table 4. Compared with responders with administrative-like missions, responders who performed missions with higher crude oil exposure opportunities were much more likely to belong to “higher-exposure” latent classes. We observed increasing monotonic dose–response relationships across latent classes for both the responders with low crude oil/mixed missions and those with crude oil-related missions.

**Table 4.** Odds ratios (OR) and 95% confidence intervals (CI) of belonging to either of the moderate exposure experience latent class (2 and 3) or high exposure class (4) versus the low exposure experience class (1), by mission category compared with the “administrative-like missions” group (n = 5,665).

Mission category	OR (95% CI) for class 2 membership versus class 1 membership	OR (95% CI) for class 3 membership versus class 1 membership	OR (95% CI) for class 4 membership versus class 1 membership
Administrative-like missions	Reference	Reference	Reference
Low oil/mixed missions	4.20 (3.17, 5.57)	6.28 (4.90, 8.05)	33.91 (25.48, 45.13)
Oil-related missions	10.12 (6.25, 16.40)	11.60 (6.25, 16.40)	57.07 (36.02, 90.40)

These results are from the latent class with covariate model constrained to have class-specific indicator response probabilities equal to those of the noncovariate measurement latent class model.

## Discussion

### Potential utility of the estimated latent classes

This latent class analysis suggests there are clustered patterns of similar exposure encountered across participants in the Deepwater Horizon Oil Spill Coast Guard Cohort Study, associated with the broad categories of mission tasks completed by Coast Guard responder personnel. These distinct patterns of exposure help distinguish more similar from less similar participants with respect to a variety of measured, as well as unmeasured, exposures, and may be useful for informing future analyses of the DWH-CG study population. We identify four clusters of the responder population distinguished by their oil/exhaust exposure, and their anxiety/time outdoors. These four groupings of similar persons may correspond to additional exposures that were not directly assessed by questionnaire or included in these models, but that might be amenable to future studies (e.g., biomarker-based exposure assessments).

### Strengths and limitations

This analysis had several major strengths. In this study population, for the six indicators used in the latent class model, there was no combination of indicators with zero observations, mitigating issues of data sparseness and boundary parameter estimates.^20^ After inclusion of these six indicators, there were a large number of additional variables that could be used in sensitivity analyses to evaluate our interpretation of the latent classes as a test for convergent construct validity.^21^ These tests for construct validity supported our interpretation of a four-class model as reflecting clustering along two major axes of exposure: exposure to crude oil and exhaust; and time outdoors/ anxiety. The use of ancillary variables as a basis for selecting substantively meaningful latent classes can help compensate for the limitations of data-driven decision rules,^22^ allowing us some flexibility around some of the likely limitations of our analysis (e.g., residual dependence of indicators given latent class). The availability of mission data (a three-level categorical variable) also allowed us to consider the reasonableness of our latent classes, again providing reassurance that a four-class model was a reasonable description of exposure patterns in this population.

This study had limitations. We did not know a participant’s timing, frequency, or location of exposures; this could lead to some clustering of participants in our analysis based on cumulative experience of ever having had exposures whose actual lived experience did not overlap much. We suspect there is variability across participants within each of the latent classes that is a function of their mission, but we were unable to stably estimate the latent classes conditional on the mission covariate without constraining the latent class model, or to test for differential measurement of indicators by the mission covariate. Common statistical decision-rules for deciding on the optimal number of latent classes such as the bootstrapped likelihood ratio test^23^ and the Lo–Mendell-Rubin likelihood ratio test^24^ rely on correct specification of dependencies between participants (e.g., no unmodeled data hierarchy, such as Coast Guard responders clustered in locations), and on conditional independence of indicators within latent class which may have been violated in this analysis (e.g., exhaust and oil exposure),^25^ so some subjective judgment is needed for selection of a preferred model. When we evaluated models by AIC,^15^ BIC,^16^ and entropy,^14^ it was to inform, but not decisively determine, the final model selection. We recommend use of the four-class model as the basis for designing future studies, based on the substantive reasonableness in light of our ancillary variables^25^ as well as the reasonableness of that model by some quantitative fit metrics (e.g., lowest BIC, similar entropy and AIC to several other models), but this recommendation is tempered by the recognition that another group of investigators may have different preferences regarding the weighting of entropy versus other considerations in model selection. Entropy favored a two-class model.

The latent classes estimated in this study are intended primarily for use in the design of future studies within this study population aiming to cost-effectively maximize interindividual variation in yet-unmeasured exposures, while minimizing confounding. Therefore, the estimated odds ratios of specific indicators given latent classes are less critical in this study than in many other latent class analyses where the goal is inference on the relationship of latent classes to other variables. These latent classes are informative about which people will be more informative to study in future study (e.g., for biomarker-based exposure assessment), but may not generalize to other external populations. This latent class model is parsimonious and excluded several exposures relevant to health but not highly prevalent in the sample (e.g., smoking). We opted for some indicators we thought might have weaker violation of the conditional independence given latent class assumption (e.g., ever using hand sanitizer, ever using sunscreen) over some others that were more prevalent but that we thought might violate the assumption more strongly. Several variables that were not used in building our latent class models, for example sleep duration, may be studied in relation to these latent classes in future study. The choice to include anxiety as an indicator in our model reflects our belief that anxiety may function as a moderator of the impact of other exposures and thus be an important dimension of composite exposures experienced by USCG responders to the oil spill.

## Conclusions

In summary, in this study, we used retrospective exposure questionnaire data to group participants in the Deepwater Horizon Oil Spill Coast Guard Cohort Study into four underlying distributions of similar exposure patterns. We hope these modeled exposure patterns will be useful for informing future investigation of health effects of exposures during the oil spill.

## Conflicts of interest statement

The authors declare that they have no conflicts of interest with regard to the content of this report.

## Supplementary Material


