# Supplementary material for: Exposure patterns among Coast Guard responders to the Deepwater Horizon Oil Spill: A latent class analysis
Source: Environ Epidemiol. 2022 May 2;6(3):e211. doi: 10.1097/EE9.0000000000000211 (PMC9187181; doi:10.1097/EE9.0000000000000211)
Supplement: Supplementary file 1 [file ee9-6-e211-s001.docx]

**SUPPLEMENTARY MATERIALS**

**Table S1: Exposure domains and affiliated candidate indicators based on the Deepwater Horizon oil spill Coast Guard Cohort survey and referring to the respondent’s deployment period.** Indicators selected for our final 6-indicator model are italicized.

| Exposure Domains | Candidate Indicators | N of smallest stratum (yes or no) |
| --- | --- | --- |
|  |  |  |
| Sleep | Sleep 1-3 hours | 111 / 5665 |
|  | Sleep 4-5 hours | 2146 / 5665 |
|  | Sleep 6-7 hours | 1841 / 5665 |
|  | Sleep 8+ hours | 582 / 5665 |
|  | Ever have insomnia | 1322 / 5665 |
|  |  |  |
| Insect and/or | Ever bitten by a fire ant | 703 / 5665 |
| animal bites | *Ever bitten by a mosquito* | *2108 / 5665* |
|  | Ever bitten by snake | 10 / 5665 |
|  | Ever bitten by spider | 493 / 5665 |
|  | Other animal/vector exposure | 508 / 5665 |
|  |  |  |
| Smoking | Ever smoke tobacco | 1068 / 5665 |
|  | Ever use smokeless tobacco | 661 / 5665 |
|  |  |  |
| Exposure to exhaust fumes or carbon monoxide / carbon monoxide, or  to crude oil | *Ever exposed to exhaust fumes or carbon monoxide / carbon monoxide* | *1937 / 5665* |
|  | Body ever came in contact with oil | 1908 / 5665 |
|  | *Ever exposed to oil* | *2755 / 5665* |
|  | Ever inhale oil vapors | 2599 / 5665 |
|  |  |  |
| Injury and | Ever have an abrasion | 380 / 5665 |
| care-seeking experiences | Ever have symptoms post deployment | 142 / 5665 |
|  | Ever experience a sprain | 175 / 5665 |
|  | Ever have puncture wound | 48 / 5665 |
|  | Ever experience a fracture | 17 / 5665 |
|  | Ever experience a laceration | 66 / 5665 |
|  | Ever request medical follow-up | 195 / 5665 |
|  |  |  |
| Anxiety and | *Ever experience anxiety* | *803 / 5665* |
| other | Ever experience bereavement/loss | 45 / 5665 |
| psychosocial | Ever depressed | 425 / 5665 |
| stressors | Ever have family issues | 267 / 5665 |
|  | Ever have fatigue | 2560 / 5665 |
|  | Ever have financial stress | 89 / 5665 |
|  | Ever have legal problems | 74 / 5665 |
|  | Ever have moral conflict | 57 / 5665 |
|  | Ever have work conflict | 173 / 5665 |
|  | Ever experience sexual abuse | 2 / 5665 |
|  | Ever have relationship issues | 347 / 5665 |
|  | Ever have substance abuse issues | 7 / 5665 |
|  | Ever have other work-life issues | 122 / 5665 |
|  | No work life issues | 395 / 5665 |
|  | Ever request worklife follow-up | 54 / 5665 |
|  |  |  |
| Use of PPE | Ever wear a Camelbak^®^ | 1082 / 5665 |
|  | Ever visit a decon. station | 757 / 5665 |
|  | *Ever use hand sanitizer* | *2113 / 5665* |
|  | Ever wear leather work gloves | 1513 / 5665 |
|  | Ever wear nitrile gloves | 2381 / 5665 |
|  | Ever use a personal flotation device | 2495 / 5665 |
|  | Ever wear protective headgear | 2451 / 5665 |
|  | Ever use bug repellant | 2421 / 5665 |
|  | Ever use a respirator | 207 / 5665 |
|  | Ever wear safety boots | 2234 / 5665 |
|  | Ever wear safety glasses | 2200 / 5665 |
|  | *Ever wear sunscreen* | *1940 / 5665* |
|  | Ever wear Tyvek^®^ suits | 642 / 5665 |
|  | Ever wear waders | 244 / 5665 |
|  | No PPE use | 1518/5665 |
|  | Ever request a safety follow-up | 31 / 5665 |

**Figure S1. Joint contingency table of the 6 selected indicators for the latent class measurement model.** The Y axis of this figure identifies the 64 possible indicator response patterns (for 6 binary indicators) based on whether a person gave a positive (1) or negative (0) response regarding having encountered each of the following exposures: any oil exposure, exhaust fumes or carbon monoxide exposure, hand sanitizer use, sunscreen use, mosquito bite, and anxiety. The X axis is the frequency of occurrence of that indicator pattern.


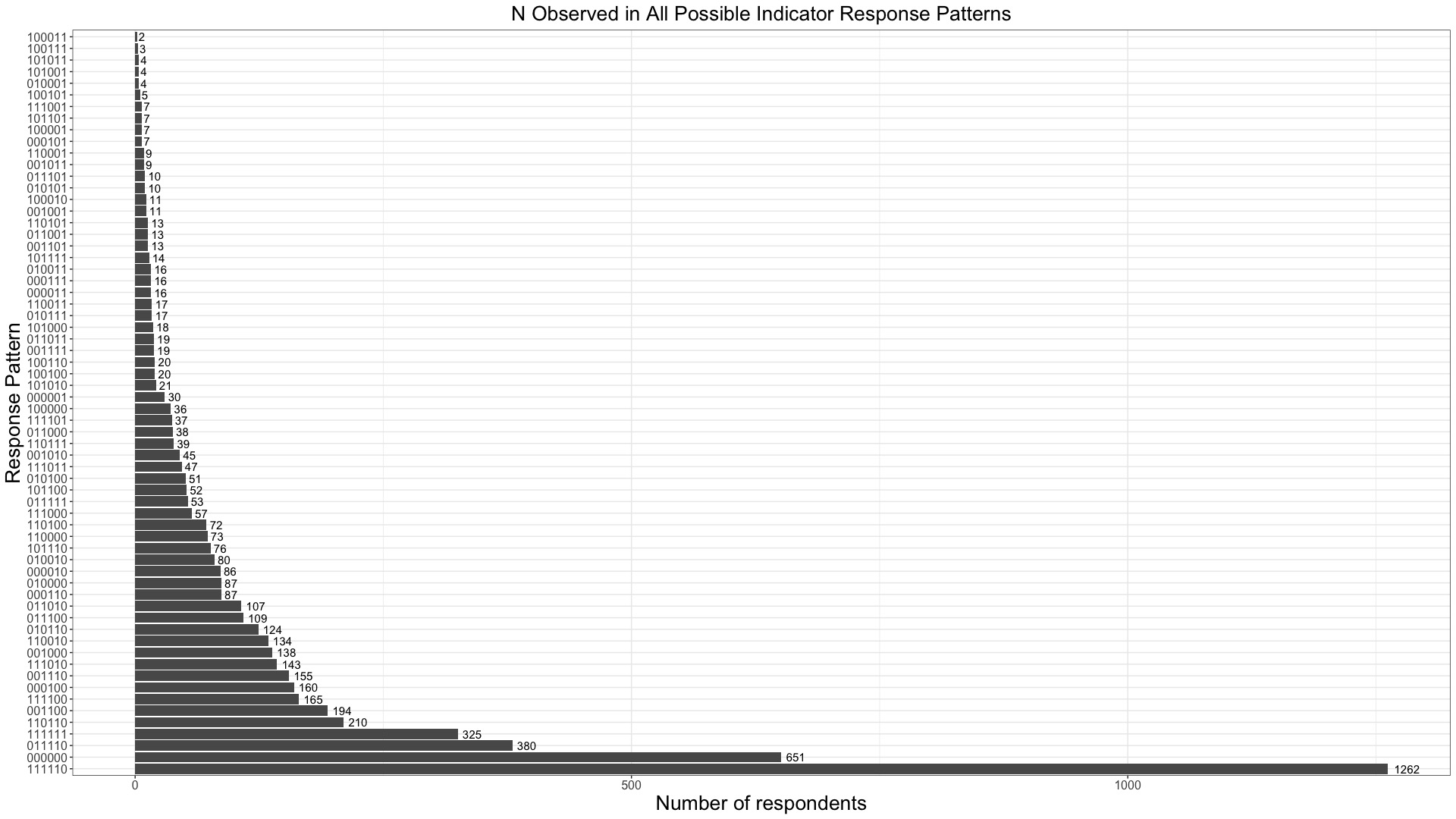


**Table S2. Exposure profiles among Coast Guard responders to the Deepwater Horizon Oil Spill, in model with classes flexibly estimated conditional on mission (n = 5,665).** Probability of self-reporting “yes” to each exposure, among members of each latent class, is shown in cells. Estimated prevalence of each latent class across the population, based on posterior probabilities, is shown in parentheses. This model was fitted using the predicted probabilities from Table 2 as starting values for class-specific indicator probabilities. Note, this flexible model had a parameter estimate at the boundary.

| Binary Exposure (Yes/No) | Class 1 (~17%) “low exposure” | Class 2 (~25%) “low oil/exhaust exposure with moderate outdoor time” | Class 3 (~15%) “high oil/exhaust exposure with moderate outdoor time” | Class 4 (~43%) “high exposure” |
| --- | --- | --- | --- | --- |
| Oil | 0.01 | 0 (boundary) | = 1 (boundary) | 0.84 |
| Exhaust fumes or carbon monoxide | 0.08 | 0.47 | 0.78 | 0.96 |
| Hand sanitizer | 0.12 | 0.59 | 0.47 | 0.91 |
| Sunblock | 0.08 | 0.67 | 0.51 | 0.94 |
| Mosquito bite | 0.07 | 0.56 | 0.55 | 0.93 |
| Anxiety | 0.05 | 0.12 | 0.15 | 0.19 |
